# Intra-operative Technique for Managing Paediatric Distal Forearm Fractures

**DOI:** 10.7759/cureus.101795

**Published:** 2026-01-18

**Authors:** Dheeraj Panchaksharam Selvarajan, Nika Majidi, Ravi Mallina

**Affiliations:** 1 Orthopaedics, Croydon Health Services NHS Trust, London, GBR

**Keywords:** fracture of distal radius, intra-operative techniques, minimally invasive surgical procedures, paediatric fracture, paediatric injuries, trauma and orthopedics

## Abstract

Paediatric distal forearm fractures are a common injury in children and adolescents, often requiring surgical intervention. Traditional techniques involve forceful manipulation and hyper-exaggerating the defect to overcome haematoma resistance, increasing the risk of complications including iatrogenic displacement, neurovascular injury, and growth plate damage. This technical note describes an alternative intra-operative reduction technique aimed at addressing these limitations.

A minimally invasive approach with a small incision allows for precise identification and gentle evacuation of the haematoma. Under image intensifier guidance, fracture fragments are carefully reduced with the use of McDonald’s elevator, utilising gentle levering and manipulation. Kirschner wires (K-wires) are then inserted percutaneously for stable fixation.

This technique offers potential technical advantages, including reduced manipulation force and minimised soft tissue disruption. Whilst formal outcome assessment is beyond the scope of this report, this approach represents a reproducible alternative for the intra-operative management of paediatric distal forearm fractures.

## Introduction

Paediatric distal forearm fractures are a prevalent injury in children and adolescents, accounting for close to two-thirds of all upper limb fractures in this age group [1]. Epidemiologic studies report a higher incidence of these fractures during summer and spring, particularly among older school-aged boys and those participating in outdoor activities [2]. As a result of this seasonal and demographic prevalence, these injuries represent a significant workload for trauma services, indicating the importance of developing efficient, safe, and reproducible management techniques.

Paediatric distal forearm fractures can present with varying complexity, from simple greenstick fractures to complex, displaced injuries that necessitate higher-level intervention. The management for this fracture type depends on numerous factors, including patient age, mechanism of injury, degree of displacement, and presence of neurovascular deficit. Treatment options range from conservative, involving closed reduction and immobilisation in the emergency department, or surgical, utilising manipulation under anaesthesia with or without Kirschner wire (K-wire) fixation [3]. The anatomy of the developing paediatric skeleton, particularly the robust periosteum and the capacity for substantial bone remodelling, presents unique challenges in management. Closed reduction and casting are typically the initial treatment of choice [4]. However, open reduction and internal fixation (ORIF) with K-wires may be indicated in cases involving significant displacement, instability, or neurovascular compromise [5].

Traditional surgical techniques utilised to address these fractures often require forceful manipulation and hyper-exaggeration of the fracture to overcome the resistance that is faced by the haematoma between the periosteum and cortex. This biomechanical resistance may limit successful reduction and necessitate repeated forceful manoeuvres, which can be troublesome, particularly with paediatric patients, increasing the risk of iatrogenic complications such as further displacement, neurovascular injury, periosteal stripping, and physeal damage [6].

This technical note aims to describe a novel intra-operative technique that addresses these biomechanical challenges by directly releasing periosteal tension and evacuating the fracture haematoma. The objective is to facilitate controlled reduction whilst minimising soft tissue trauma. Based on our clinical experience, this technique is best suited for acute paediatric distal forearm fractures, typically within the first 7-10 days following injury, when the fracture haematoma remains present and the periosteal sleeve is intact but not yet organised. Beyond this period, progressive haematoma consolidation and early callus formation may reduce the effectiveness of haematoma evacuation and periosteal release.

## Technical report

**Figure 1:**
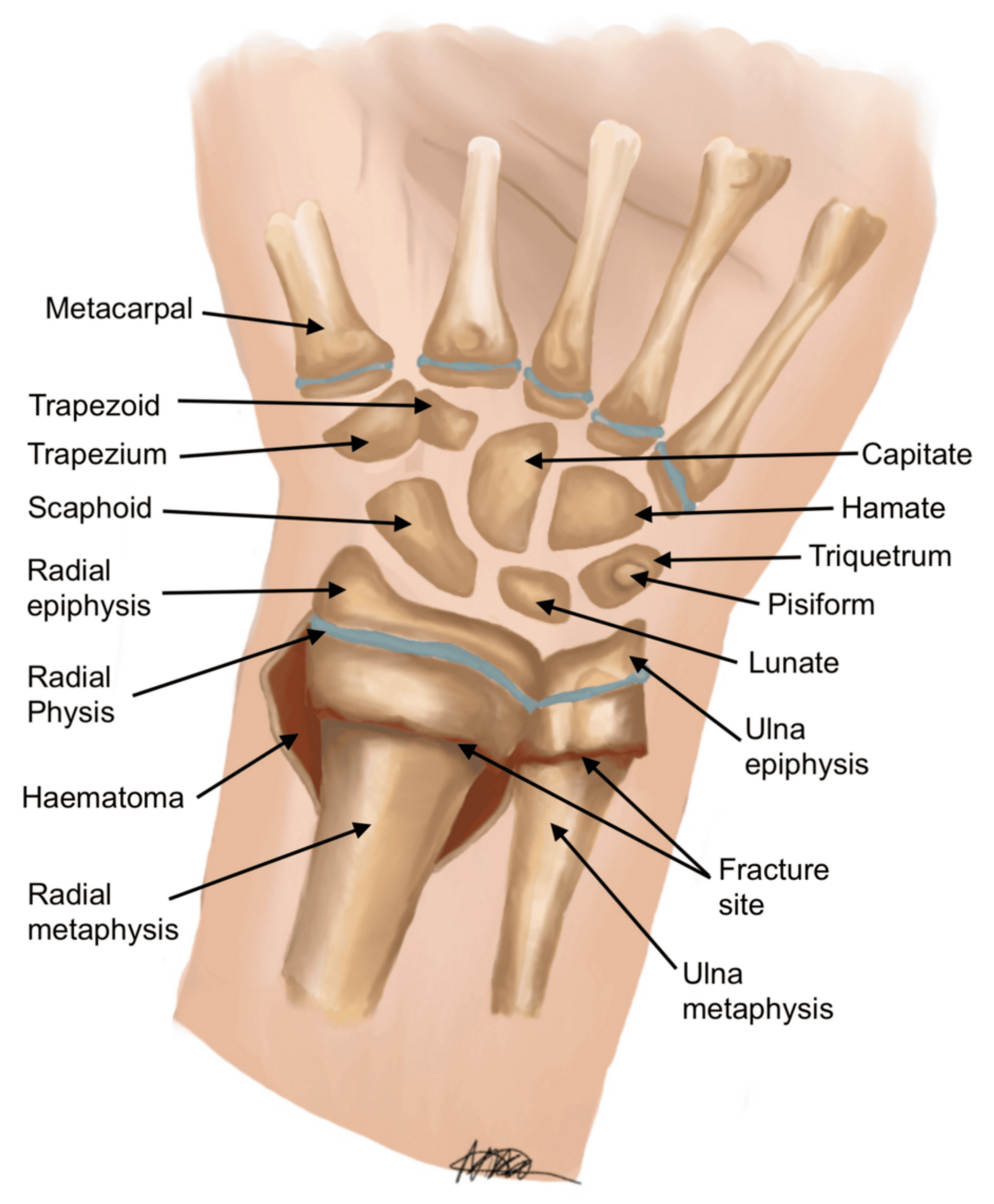
Posteroanterior (PA) view of the distal radius and ulna fracture, displaced dorsally in a skeletally immature patient, with intact periosteum, cortex, and haematoma outlined separately Image credit: Nika Majidi

**Figure 2:**
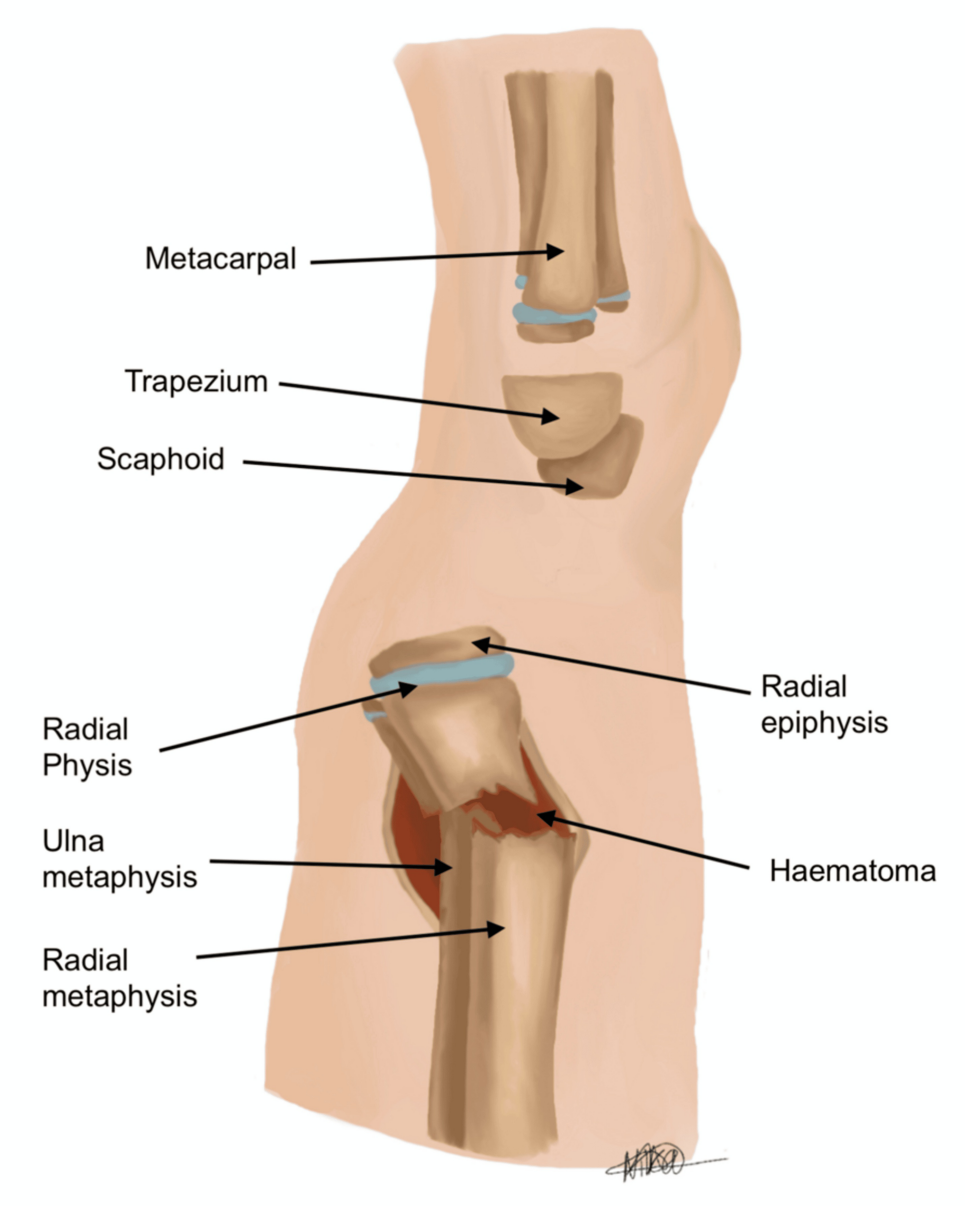
Lateral view of distal radius and ulna, with intact periosteum, cortex, and haematoma Image credit: Nika Majidi

Intra-operative challenges of reduction and K-wire fixation at the level of metaphysis are fraught with technical challenges due to the acute transition of a broad metaphysis into the diaphysis. This is compounded by difficulty in achieving a satisfactory closed reduction with traditional reduction manoeuvres. Figures 1, 2 indicate the posteroanterior (PA) and lateral views, respectively, of a dorsally displaced distal radius and ulna fracture in an immature skeleton. As evidenced in the diagram, there is an intact periosteum, cortex, and surrounding haematoma, which can further complicate reduction efforts.

**Figure 3:**
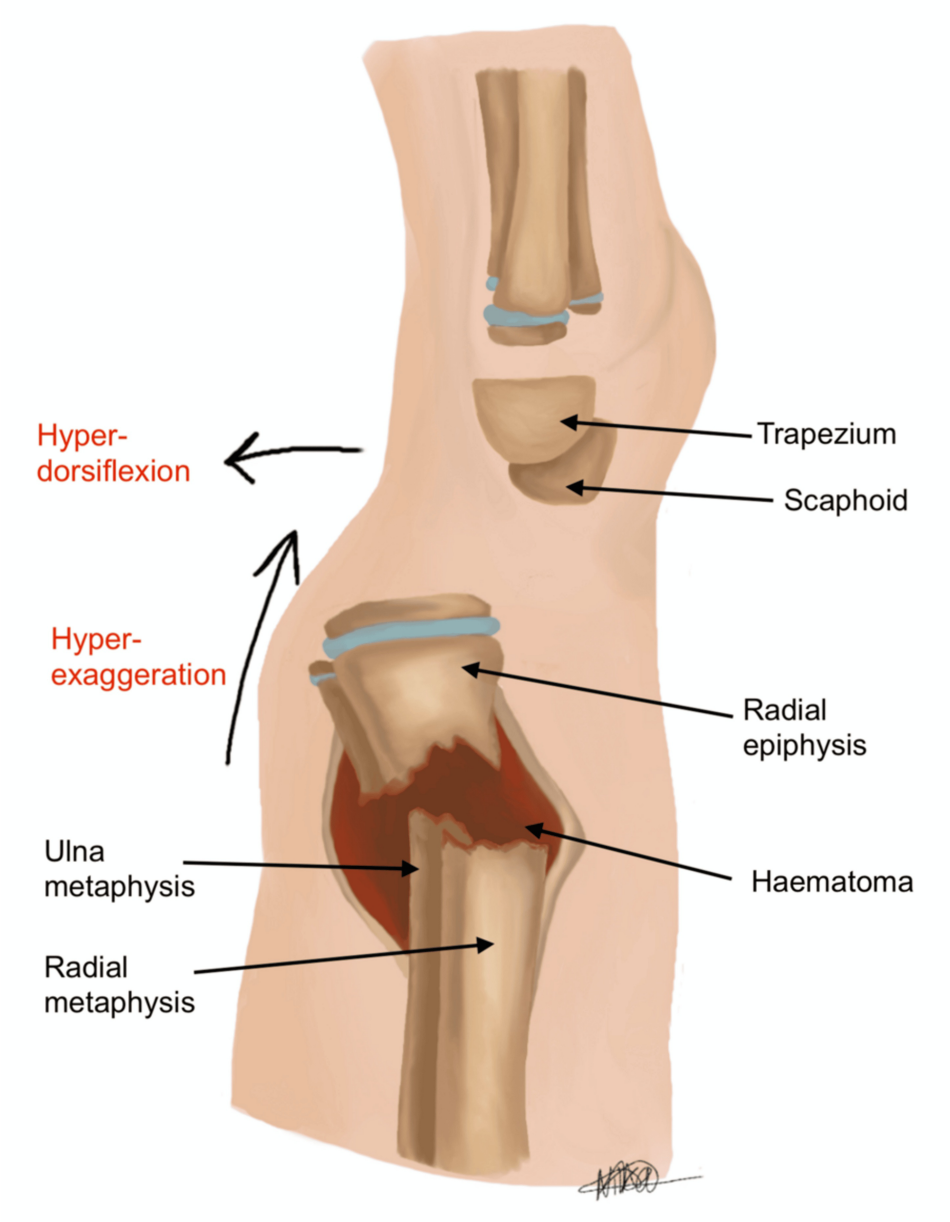
Lateral view showing hyper-exaggeration of the fracture deformity, but with intact periosteum and underlying haematoma Image credit: Nika Majidi

**Figure 4:**
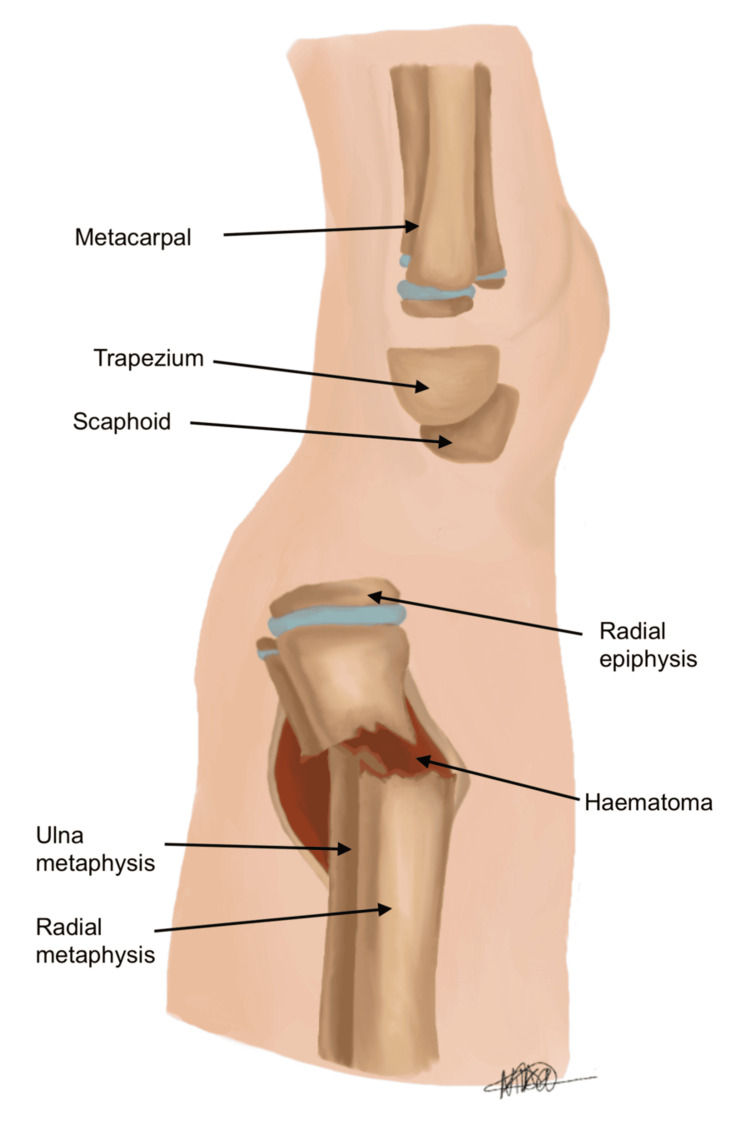
Lateral view, post hyper-exaggeration, no satisfactory reduction of fracture fragments Image credit: Nika Majidi

Typically, the classical method of reduction involves hyper-exaggeration and hyper-dorsiflexion of the wrist (see Figure 3) [7], with the aim of subsequently reducing the fracture into an anatomical position. This is followed by cast immobilisation, or if intra-operatively deemed unstable, K-wire fixation is performed. However, this approach relies on overcoming resistance indirectly and may be limited by the intact periosteal sleeve and haematoma, resulting in incomplete reduction, as demonstrated in Figure 4. Repeated or forceful manipulation may further increase the risk of iatrogenic injury, particularly in the paediatric population. We herein describe our novel technique for treating these fractures, which emphasises minimal force and controlled reduction.

**Figure 5:**
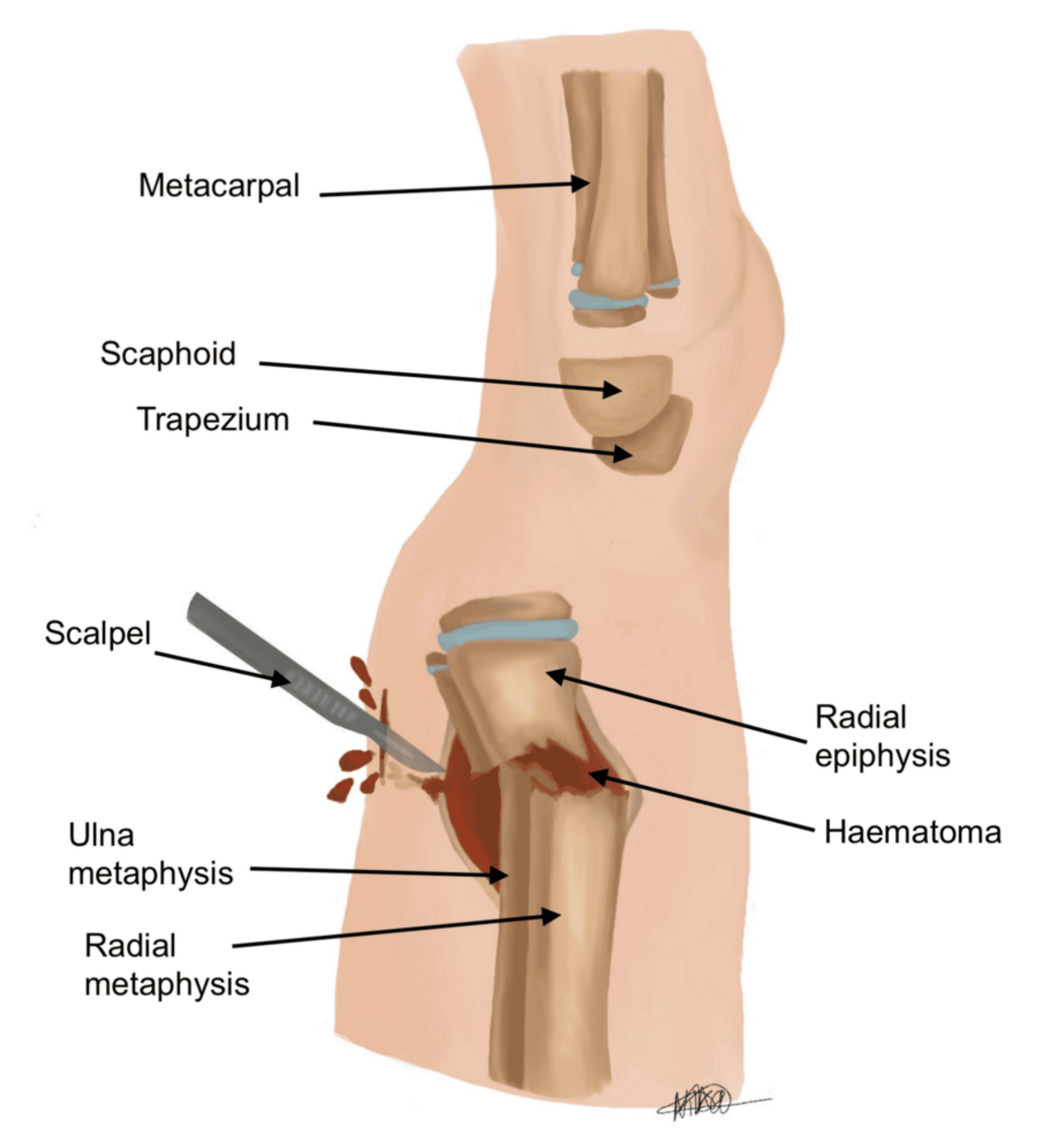
Lateral view, 0.5 cm stab incision over the periosteum, around the level of fracture haematoma, evacuating haematoma and de-tensioning the periosteal layer Image credit: Nika Majidi

**Figure 6:**
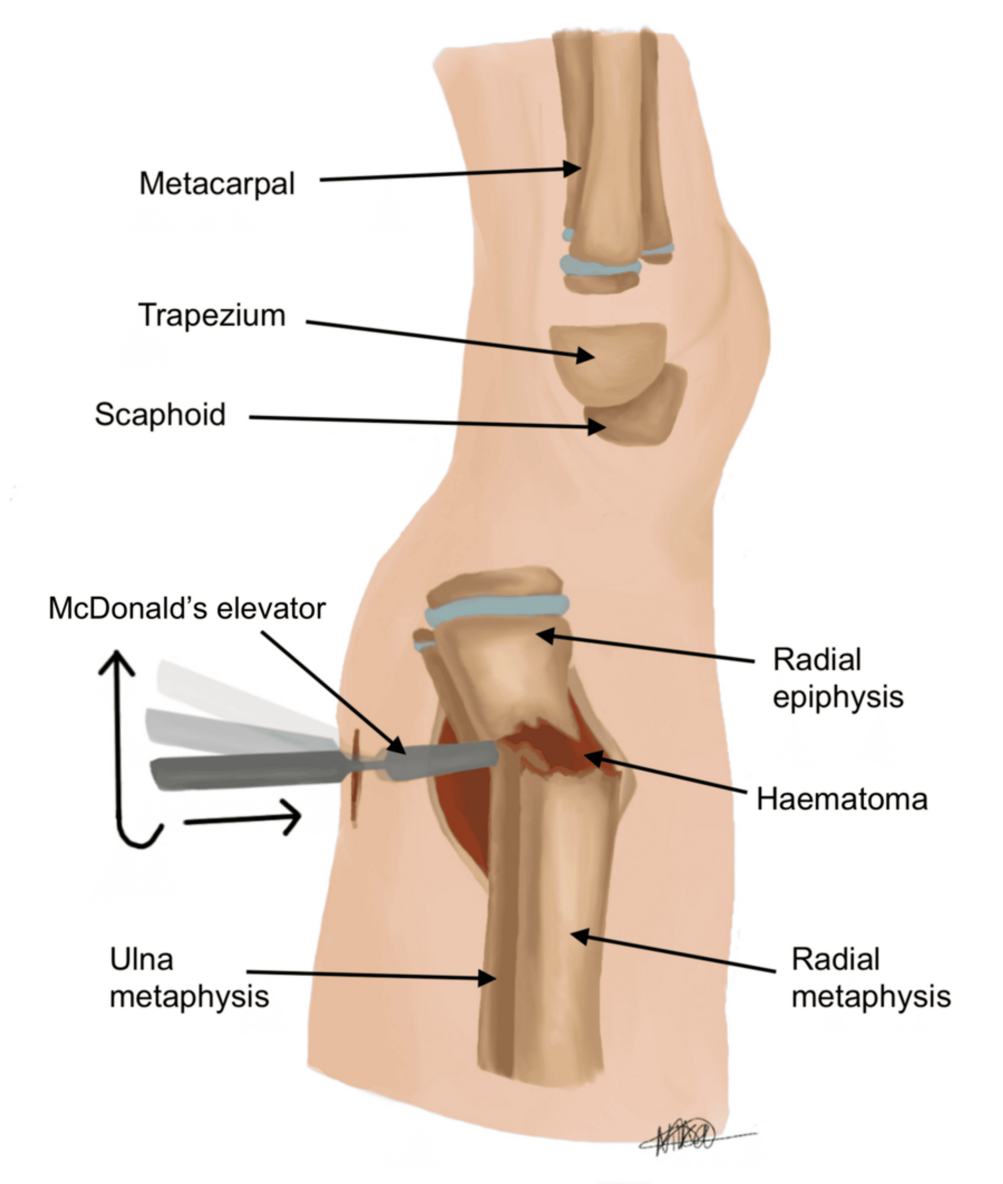
Lateral view, insertion of McDonald's elevator at fracture site, levering the distal fragment to achieve satisfactory reduction Image credit: Nika Majidi

**Figure 7:**
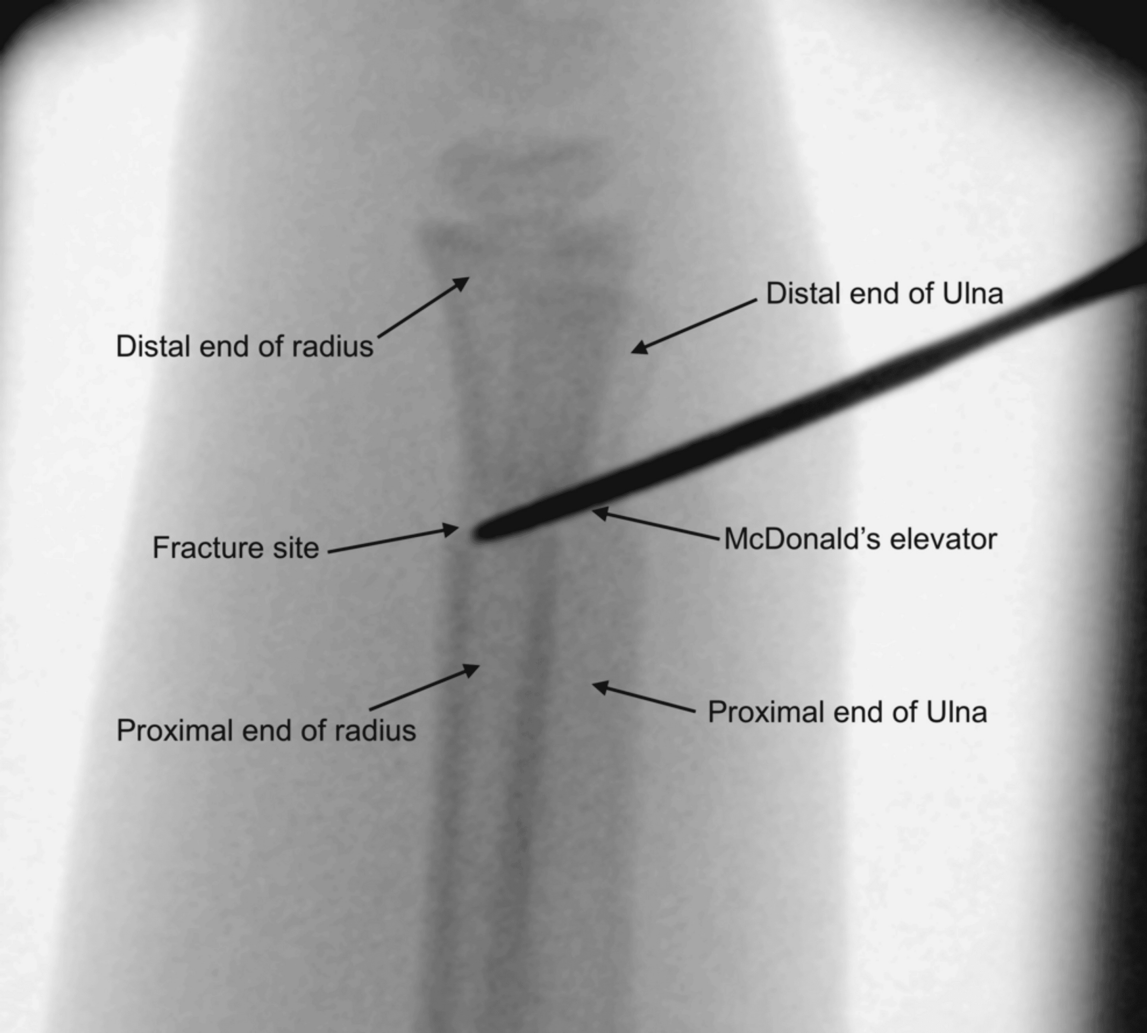
Fluoroscopic image showing lateral view of intra-operative technique of using McDonald's elevator to lever the distal fragment over the proximal fragment

**Figure 8:**
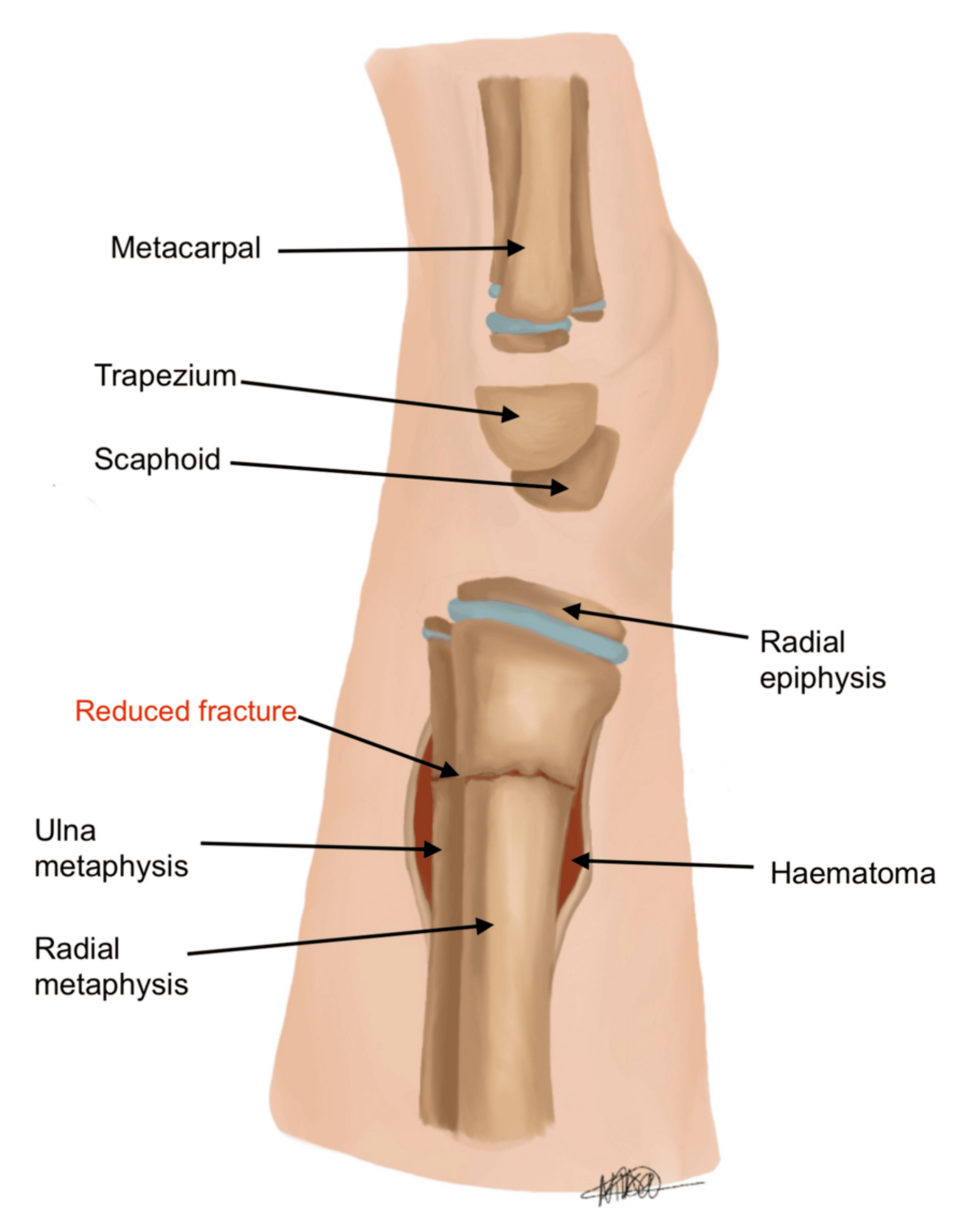
Lateral view, reduction of fracture deformity Image credit: Nika Majidi

**Figure 9:**
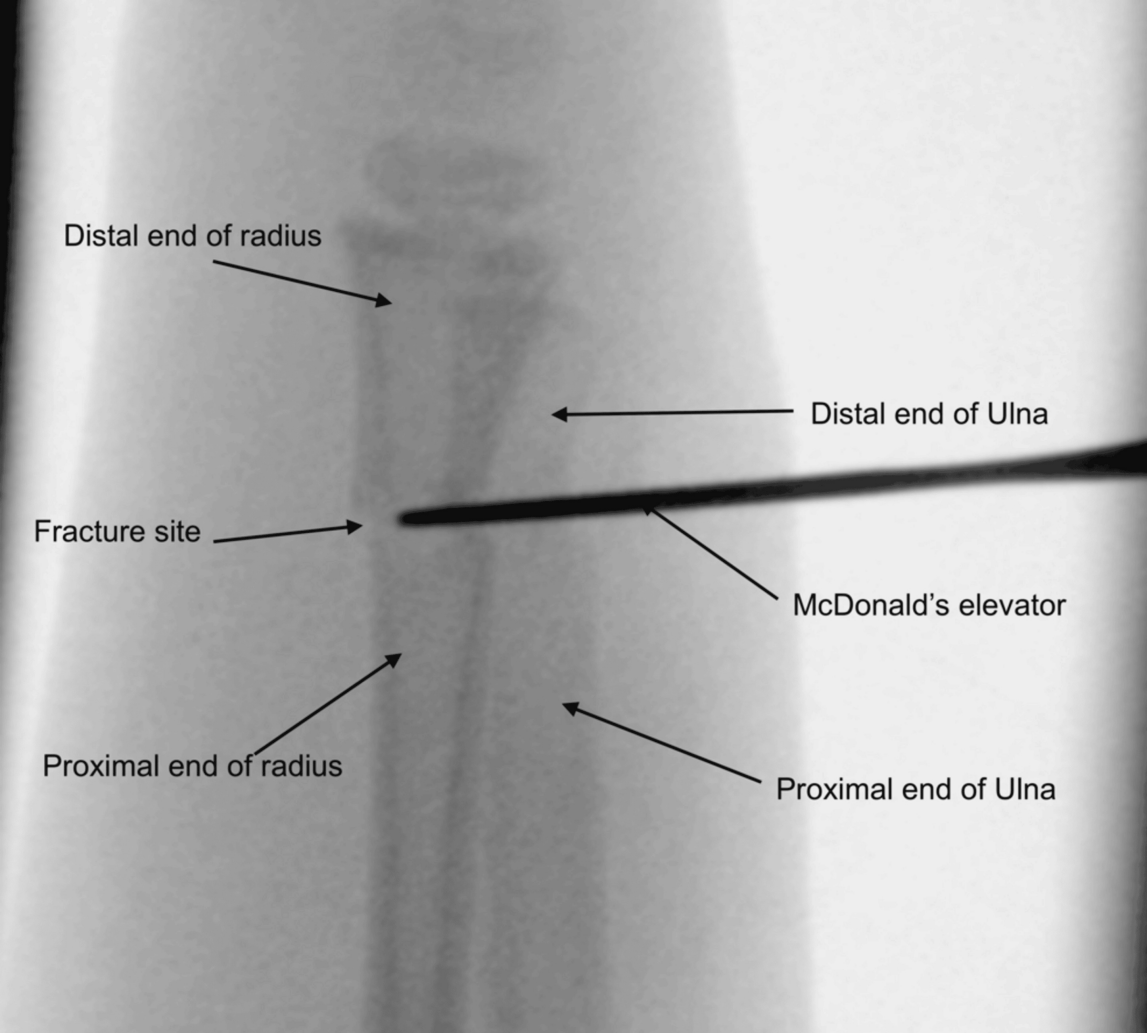
Intra-operative image (lateral view) showing alignment of fracture fragments with McDonald's elevator

Our technique differs by directly addressing the source of resistance. Utilising image guidance, the fracture fragments are reduced under direct visualisation on the lateral view. A stab incision (approximately 0.5) is performed over the periosteum, around the level of the fracture haematoma, as illustrated in Figure 5. The incision is placed on the dorsal aspect of the distal forearm directly over the fracture apex, as localised fluoroscopically in the lateral plane. The haematoma is then gently evacuated to release periosteal tension, allowing for improved mobility of the fracture fragments. Following this, McDonald’s elevator is introduced at the fracture site, and the distal fragment is gently levered into place to achieve satisfactory reduction (see Figures 6, 7). The instrument is advanced in a controlled subperiosteal plane, with minimal depth of dissection, under fluoroscopic guidance to reduce the risk of injury to surrounding soft tissues. Particular attention is paid to avoiding excessive force that could compromise the periosteum or adjacent neurovascular structures, including the superficial radial sensory nerve. This controlled levering technique avoids the need for hyper-exaggeration of the deformity, achieving reduction with minimal force. Figures 8, 9 show the illustration and intra-operative radiograph, respectively, of the successful reduction using this technique.

In comparison, the traditional method uses exaggeration of the deformity to overcome resistance, whereas our approach eliminates this resistance through releasing the haematoma and periosteal tension. This novel method has various benefits, including a more predictable reduction and decreased manipulation force. We therefore predict that this method is likely to have a reduced risk of iatrogenic injury. Additionally, through this technique, fewer attempts at reduction are likely needed, thereby shortening operative time.

**Figure 10:**
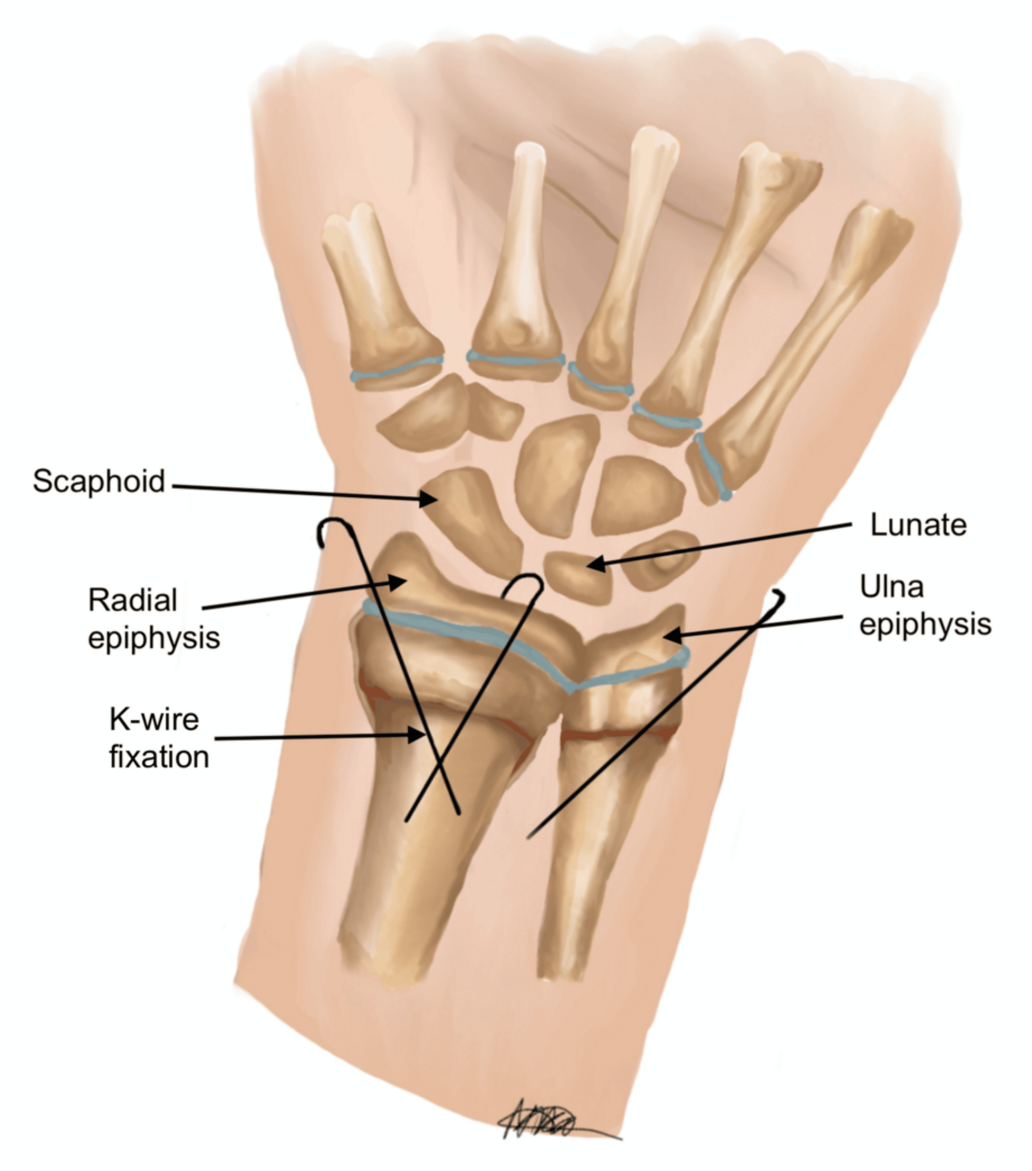
Posteroanterior (PA) view, reduction of fracture deformity, and placement of Kirschner wires (K-wires) Image credit: Nika Majidi

**Figure 11:**
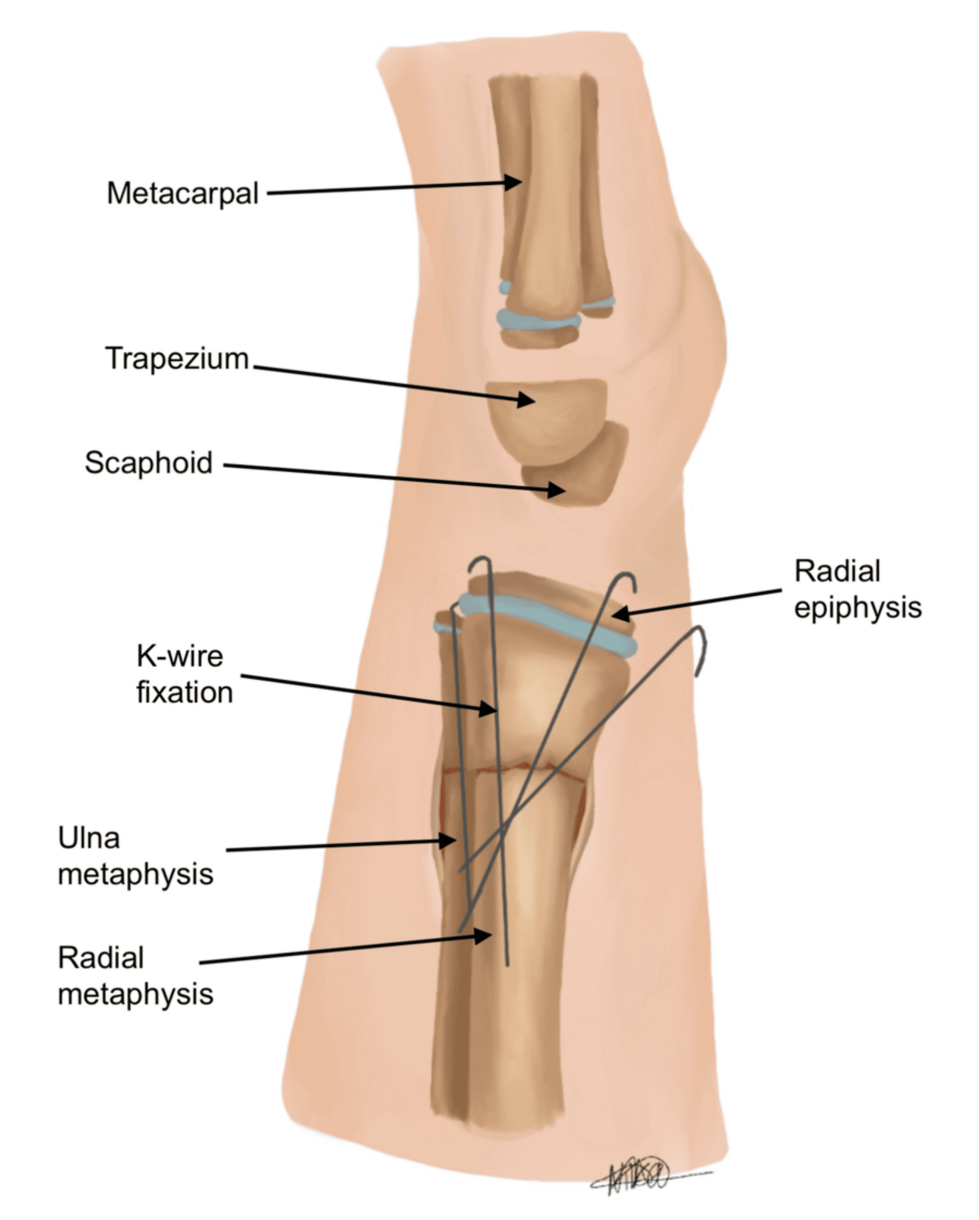
Lateral view of reduction of deformity and placement of Kirschner wires (K-wires) Image credit: Nika Majidi

Finally, to ensure optimal placement and stability, K-wires were inserted percutaneously under fluoroscopic guidance. As with all K-wire fixation, careful attention should be paid to avoid iatrogenic injury to neurovascular structures. Figures 10, 11 show the final PA and lateral views of the successful reduction of the fracture deformity and fixation with K-wires.

## Discussion

A significant proportion of paediatric distal forearm fractures can be treated effectively with closed reduction and casting. In cases of unsuccessful reduction, significant displacement, instability, or more complex fractures, surgical fixation is commonly warranted. Younger children typically have more acceptable results through closed reduction compared to older patients due to open growth plates allowing for increased remodelling capacity [8]. Therefore, this minimises the requirement for surgical intervention in this age group. However, research indicates this remodelling capacity is greatly reduced in children over 10 years old, increasing the requirement for precise anatomical reduction, frequently necessitating surgical intervention to ensure appropriate alignment and long-term functional outcomes [9,10].

Conventional closed reduction and casting typically leads to favourable outcomes. However, there are limitations to this approach. There has been a reported 29% failure rate using closed reduction in the treatment of cases with more significant displacement, with fractures of angulation over 15 degrees and over 45 degrees of malrotation having higher risks of suboptimal healing or long-term functional impacts [9,11].

The operative treatment of these fractures has experienced substantial growth over the past years [9]. The purpose of this technical report is not to compare outcomes but to describe an alternative reduction strategy that addresses a recognised limitation of traditional methods. Our surgical technique emphasises a more gentle and controlled approach to fracture reduction, using a minimally invasive exposure (less than 1 cm stab incision) with minimal soft tissue disruption. Traditional closed reduction methods often require hyper-exaggeration of the deformity, which is associated with increased risk of complications, including iatrogenic displacement, neurovascular injury, and physeal damage [7]. In comparison, our method involves sharp dissection to identify fracture fragments, followed by gentle haematoma evacuation. This aims to reduce resistance to reduction, allowing for accurate and more gentle fragment manipulation with McDonald's elevator.

The importance of minimising the force used in the manipulation of paediatric fractures has been well-documented. Forceful manipulation during the process of reduction can lead to periosteal stripping and disruption of the growth plate, thereby potentially adversely affecting long-term outcomes [9]. Our technique importantly mitigates these risks by leveraging controlled manipulation using McDonald's elevator. Image guidance also plays a vital role in our technique, ensuring real-time assessment of reduction quality and avoidance of excessive force, alongside reducing the risk of iatrogenic injury. In a review of literature, minimally invasive approaches have gained popularity in paediatric fracture management due to their benefits with diminished soft tissue damage, lower infection rates, and faster rehabilitation times [2]. Our described approach aligns with these principles, ensuring optimal placement and stability whilst minimising iatrogenic injury to neurovascular structures and the physis. Furthermore, Korup et al. emphasised the increasing incidence of paediatric forearm fractures and the necessity for techniques that balance efficacy and safety [1]. Our method addresses this by providing a reproducible, less traumatic alternative to traditional closed reduction methods.

This report is limited by its presentation of an illustrative case and the absence of quantitative outcome data. Formal evaluation of operative time, radiation exposure, complication rates, and functional outcomes was beyond the scope of this technical note. Nevertheless, based on intra-operative experience and the mechanistic rationale of the technique, several potential advantages over standard reduction methods are hypothesised. These include reduced operative time due to fewer attempts at manipulation, decreased fluoroscopy exposure secondary to a more predictable reduction, and a lower risk of iatrogenic soft tissue or physeal injury by avoiding forceful hyper-exaggeration manoeuvres. Additionally, direct evacuation of the fracture haematoma and release of periosteal tension may reduce the likelihood of incomplete reduction or the need for repeat manipulation. These limitations are acknowledged, and a prospective case series with comparative outcome analysis is planned as the next stage of investigation.

## Conclusions

We present a technical report describing an alternative intra-operative reduction technique for a paediatric distal radial fracture managed at a District General Hospital in London. Post-operative management consisted of pin-site review at one week, K-wire removal at four weeks, and subsequent immobilisation in a below-elbow cast.

This report demonstrates the technical feasibility and reproducibility of a controlled reduction strategy that directly addresses periosteal tension and haematoma resistance. Whilst no outcome conclusions can be drawn from a single case, this technique may offer a useful adjunct in cases where traditional closed reduction proves difficult. Further prospective studies are required to evaluate clinical outcomes and comparative effectiveness.
